# Reliability of Tibialis Anterior Muscle Voluntary Activation Using the Interpolated Twitch Technique and the Central Activation Ratio in People with Stroke

**DOI:** 10.3390/brainsci11020176

**Published:** 2021-02-01

**Authors:** Sharon Olsen, Nada Signal, Imran Khan Niazi, Gemma Alder, Usman Rashid, Rasmus Bach Nedergaard, Denise Taylor

**Affiliations:** 1 Health and Rehabilitation Research Institute, Auckland University of Technology, Auckland 0627, New Zealand; nada.signal@aut.ac.nz (N.S.); gemma.alder@aut.ac.nz (G.A.); usman.rashid@aut.ac.nz (U.R.); denise.taylor@aut.ac.nz (D.T.); 2 Centre for Chiropractic Research, New Zealand College of Chiropractic, Auckland 1060, New Zealand; imran.niazi@nzchiro.co.nz; 3 Mech-Sense, Department of Gastroenterology and Hepatology, Aalborg University Hospital, 9000 Aalborg, Denmark; r.nedergaard@rn.dk; 4 Department of Clinical Medicine, Aalborg University, 9000 Aalborg, Denmark

**Keywords:** voluntary activation, interpolated twitch, central activation ratio, stroke, reliability

## Abstract

Voluntary activation (VA) is measured by applying supramaximal electrical stimulation to a muscle during a maximal voluntary contraction (MVC). The amplitude of the evoked muscle twitch is used to determine any VA deficit, and indicates incomplete central neural drive to the motor units. People with stroke experience VA deficits and greater levels of central fatigue, which is the decrease in VA that occurs following exercise. This study investigated the between-session reliability of VA and central fatigue of the tibialis anterior muscle (TA) in people with chronic stroke (*n* = 12), using the interpolated twitch technique (ITT), adjusted-ITT, and central activation ratio (CAR) methods. On two separate sessions, supramaximal electrical stimulation was applied to the TA when it was at rest and maximally activated, at the start and end of a 30-s isometric dorsiflexor MVC. The most reliable measures of VA were obtained using the CAR calculation on transformed data, which produced an ICC of 0.92, and a lower bound confidence interval in the good range (95% CI 0.77 to 0.98). Reliability was lower for the CAR calculation on non-transformed data (ICC 0.82, 95% CI 0.63 to 0.91) and the ITT and adjusted-ITT calculations on transformed data (ICCs 0.82, 95% CIs 0.51 to 0.94), which had lower bound confidence intervals in the moderate range. The two ITT calculations on non-transformed data demonstrated the poorest reliability (ICCs 0.62, 95% CI 0.25 to 0.74). Central fatigue measures demonstrated very poor reliability. Thus, the reliability for VA in people with chronic stroke ranged from good to poor, depending on the calculation method and statistical analysis method, whereas the reliability for central fatigue was very poor.

## 1. Introduction

Voluntary activation (VA) is a measure of the voluntary neural drive to a muscle during a voluntary contraction [1]. Its measurement involves the application of supramaximal electrical stimulation to a motor nerve or muscle during a maximal voluntary contraction (MVC) [2]; the twitch force produced by this electrical stimulation indicates the proportion of muscle activation that is not under voluntary control [1]. VA deficits indicate incomplete activation of motor units due to insufficient recruitment or discharge rate [3], and can arise from dysfunction at any point proximal to the location of electrical stimulation, including in the cortex, subcortical areas, descending tracts, or motor axons [4]. Following stroke, VA deficits have been observed in the hemiparetic elbow flexors [5,6,7], knee extensors [8,9,10], ankle plantarflexors [11], and ankle dorsiflexors [12,13]. These deficits in VA are likely due to the reduction in corticomotor excitability that accompanies stroke [14,15].

The methods used to record and quantify VA vary across the literature. Electrical stimulation is applied supramaximally over either a peripheral nerve or muscle, using a single, doublet, or train of pulses [16]. While a train of pulses provides greater activation of the muscle [17], single or doublet pulses are less likely to produce antidromic activation of motor neurons and Renshaw cells, or reflex synergistic activation [4] and may also be preferred for comfort [18]. VA can be calculated in two ways: the interpolated twitch technique (ITT) or the central activation ratio (CAR) [16]. Both involve the application of supramaximal electrical stimulation to the motor nerve or muscle while the target muscle is performing an MVC. If the muscle is not already maximally activated, this stimulation will produce additional muscle force (interpolated twitch) [1]. The ITT calculation involves expressing the interpolated twitch as a proportion of the twitch force produced when same stimulation is applied to the resting muscle [1]. The CAR method involves expressing the amplitude of the MVC just prior to the interpolated twitch as a proportion of the total force produced by the MVC plus the interpolated twitch [17]. In general, the ITT method is employed when a single or doublet pulse is used, while the CAR method is employed when a train of stimulation is used [16,17,18]. However, both ITT and CAR calculations have been employed with doublet pulses to measure VA [18,19,20]. VA can also be measured via the application of transcranial magnetic stimulation (TMS) over the cortex [21]; however, this technique will not be addressed in this paper.

Following a bout of high-intensity exercise, decreases in VA are observed [4,20]. This phenomenon is known as central fatigue and represents suboptimal output from spinal and supraspinal circuitry [4]. Central fatigue can be determined by calculating the change in VA from immediately before to immediately after the exercise [20]. This central source of neuromuscular fatigue is more prominent in the hemiparetic limb following stroke [7,13].

VA measurements provide a method to indirectly determine the neural drive to a muscle [4] and have therefore been used to gauge the effects of interventions such as neuromuscular electrical stimulation [22,23], intramuscular needling [24], endurance training in people with multiple sclerosis [25], and neuromodulatory interventions, in healthy participants [26,27,28,29,30] and participants with stroke [31,32]. A number of studies have explored the reliability of VA measures in the healthy knee extensors [20,33,34,35,36,37,38,39] and ankle plantar flexors [40,41,42,43,44], using either the ITT [34,37,38,39,41,42,43,44,45], CAR [33,35,36,40], or a combination of both methods [20], and the majority have demonstrated good within- or between-session reliability (ICCs > 0.8 [36,37,38,40,41,42,43] or coefficients of variation <5% [20,33,34,39]). However, despite a reasonable body of evidence investigating VA in the healthy population, only one study has investigated the reliability of VA in people with stroke, in this case of the hemiparetic quadriceps muscle [46]. We are not aware of any studies that have tested the reliability of VA of the hemiparetic tibialis anterior (TA) muscle. This paper investigates the between-session reliability of VA and central fatigue of the TA muscle in people with stroke, using both the CAR and ITT methods. The focus is on the primary ankle dorsiflexor muscle, due to its key influence on gait impairment and loss of functional mobility after stroke [47,48,49,50,51] and because it is commonly targeted by rehabilitation interventions.

## 2. Materials and Methods

### 2.1. Study Design

This observational study was nested within a repeated-measures cross-over experiment that explored the effects of a neuromodulatory intervention on measures of muscle strength and neurophysiology [32]. The original cross-over experiment analyzed changes in measures of VA and central fatigue following a single session of endogenous paired associative stimulation (ePAS) versus a single session of a sham intervention [32], where the order of the intervention was random. The baseline measures that were collected seven days apart have been utilized in the following repeated-measures reliability study. In contrast to the previous “cross-over” study, the data here is grouped in chronological order (i.e., Test 1 vs. Test 2).

### 2.2. Participants

Inclusion criteria specified that participants should be over 18 years of age, with a single stroke more than 6 months prior, and hemiparesis affecting ankle dorsiflexion movement. Criteria for exclusion were significant cognitive, perceptual, or communication deficits (assessed according to the ability to follow verbal and visual commands to move the hemiparetic limb, assessed by the screening Physiotherapist), cerebellar stroke, contra-indications to peripheral electrical stimulation, an inability to generate ankle dorsiflexor force, or medical conditions that would impact the safety of the participant or their ability to complete the protocol. The sample size was 15.

### 2.3. Procedures

Participants had been asked to refrain from exercise on the day of testing. They were seated with the hemiparetic leg positioned in a purpose-built dynamometer (described previously in [32]) with a fixed ankle plate angled 25° from horizontal (plantarflexion). Following skin preparation, two EMG surface electrodes (Blue sensor N, Ambu, Ballerup, Denmark) were placed over the hemiparetic TA, a third of the way along the line between the head of the fibula and the tip of the medial malleolus [52], and a third electrode was placed on the lower third of the anterior border of the tibia.

**Figure 1:**
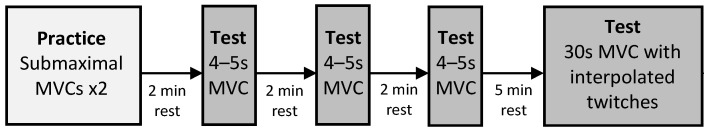
Session flow (replicated in second session).

**Figure 2:**
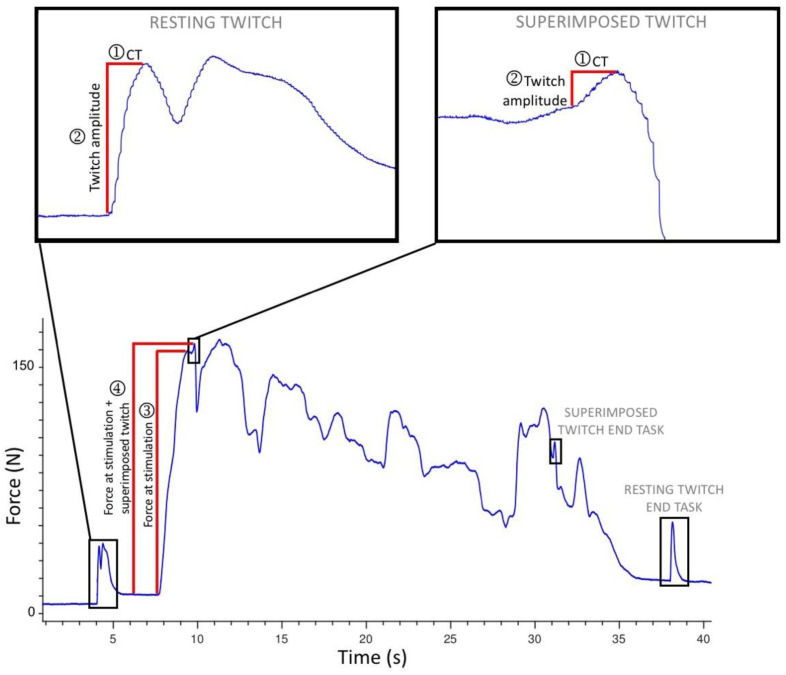
Force trace showing 30-s isometric maximal voluntary contraction (MVC) task for an individual participant, with resting and superimposed twitches identified at the start and end of the task for the calculations of voluntary activation (VA) and central fatigue. CT = contraction time.

Following two submaximal practices and a two-minute rest, participants performed three 4 to 5-s isometric MVCs (with no stimulation), with two-minute rests in between (refer to Figure 1). Participants were instructed to “pull as fast and hard as possible” and received loud verbal encouragement and real-time visual feedback of their force production. In preparation for applying interpolated twitches, two muscle stimulation electrodes (5 × 5 cm PALS, Axelgaard, Fallbrook, CA, USA) were placed over the hemiparetic TA muscle, just below the tibial plateau, and approximately midway down the tibia. To determine the optimal electrode position, single 1-ms pulses of electrical stimulation (DS7A, Digitimer Ltd., Hertfordshire, UK) were applied to the TA muscle at increasing intensity until a twitch contraction was palpable at the tendon of the TA muscle, without concurrent twitches in the tendons of the peroneal, plantar flexor, or toe extensor muscles. The location of the electrodes was marked on the skin with an indelible pen to ensure the electrode positions were repeatable. To determine the intensity of electrical muscle stimulation, doublet 1-ms pulses (10-ms inter-pulse interval, 300 V) were applied to the resting TA muscle in increasing 5-mA increments until a plateau in twitch force was reached [33,34,44]; the intensity of stimulation was set at 120% of this value. The maximum tolerated intensity was used for two participants who could not tolerate higher intensities of stimulation (24). Following a five-minute rest period, participants completed a single 30-s isometric MVC while receiving loud continuous verbal encouragement and real-time visual feedback. Using manual triggering, doublet 1-ms pulses (10-ms inter-pulse interval, 300 V) were applied to the TA during the initial resting period (resting twitch), at the start of the MVC task once a plateau in force had been reached (superimposed twitch), at the end of the fatigue task (superimposed twitch end task), and after task completion (resting twitch end task) (refer to Figure 2). Force and TA EMG data were collected simultaneously during the three MVCs and the 30-s MVC task. Force signals were amplified (with an adjustable gain of 200, 500, or 1000 depending on amplitude) (Forza, OT Bioelettronica, Torino, Italy) and sampled at 1961 Hz using a data acquisition board (Micro 1401, CED, Cambridge, UK) and Spike2 software (CED, Cambridge, UK). TA EMG data was amplified (×500) (AMT-8, Bortec Biomedical, Calgary, AB, Canada), then sampled at 1961 Hz using a data acquisition board (Micro 1401, CED, Cambridge, UK) and Spike2 software (CED, Cambridge, UK). Procedures were replicated for the second session.

### 2.4. Data Processing

Data processing was performed using Spike2 software (CED, Cambridge, UK) and Microsoft Excel software (version 16.35, Microsoft Corporation, Redmond, WA, US). The peak amplitude of the three brief MVCs [53] was measured in Spike2, and the mean was calculated in Microsoft Excel. In Spike2, for the 30-s MVC task, the resting and superimposed muscle twitches in the force data were identified visually and the following parameters were extracted (see Figure 2): (1) twitch contraction time (CT), (2) twitch peak to peak amplitude, (3) amplitude of the MVC just prior to the delivery of superimposed twitches (force at stimulation), and (4) total force produced by the MVC plus the superimposed twitches (force at stimulation + superimposed twitch). If there was any uncertainty about the onset of the twitch or its duration, the assessor considered the biologically feasible twitch duration and latency [4,19,54], to ensure the twitch was measured, and not any pre- or post-twitch muscle activity. This occurred on occasion when there was a voluntary contraction or involuntary muscle spasm immediately following the resting muscle twitch, resulting in a second visible twitch on the force data. In the case where the interpolated twitch was delivered over a rising or descending force signal and the twitch onset was unclear, the conduction time (from EMG artefact to twitch onset) of another twitch for that participant was used to define the twitch onset.

Using Microsoft Excel, VA was calculated using the ITT method (VA^ITT)^ [1], an adjusted ITT method (VA^Adj_ITT^) which accounts for variations in force at the time of stimulation [43,55], and the CAR method (VA^CAR^) [17] (see calculations below).

$${\mathrm{VA}}^{\mathrm{ITT}}\;=\;\left(1\;-\;\frac{\mathrm{Superimposed}\;\mathrm{twitch}\;\mathrm{amplitude}}{\mathrm{Resting}\;\mathrm{twitch}\;\mathrm{amplitude}}\right)\;\times \;100$$

$${\mathrm{VA}}^{\mathrm{Adj}\_\mathrm{ITT}\;}=\;\left(1-\;\frac{\mathrm{Superimposed}\;\mathrm{twitch}\;\mathrm{amplitude}\;\times \;\frac{\mathrm{Force}\;\mathrm{at}\;\mathrm{stimulation}}{\mathrm{Mean}\;\mathrm{of}\;3\;\mathrm{MVCs}}}{\mathrm{Resting}\;\mathrm{twitch}\;\mathrm{amplitude}}\right)\;\times \;100$$

$${\mathrm{VA}}^{\mathrm{CAR}}\;=\;\left(\;\frac{\mathrm{Force}\;\mathrm{at}\;\mathrm{stimulation}}{\mathrm{Force}\;\mathrm{at}\;\mathrm{stimulation}\;+\;\mathrm{Superimposed}\;\mathrm{twitch}}\right)\;\times \;100$$

VA was also calculated at the end of the 30-s task, using the ITT and CAR methods. The difference between the two VA measures at the start and end of the task was defined as central fatigue (CF^ITT^ and CF^CAR^) [20].

### 2.5. Statistical Analysis

All parameters and calculations were descriptively analysed (mean ± standard deviation (SD)) in Microsoft Excel. Data for individual twitch amplitudes, VA^ITT^, VA^Adj_ITT^, VA^CAR^, CF^ITT^, and CF^CAR^ were imported into R (Version 3.6.3, R Foundation for Statistical Computing, Vienna, Austria, 2020) for the reliability analysis. The normality of the variables was assessed with the Shapiro–Wilk test. For variables which were normally distributed, a linear mixed model with Gaussian distribution and identity link was setup to estimate the between-participant, between-test, and error (within-participant) variance using the *rptR* package [56]. To determine relative between-session reliability, the intraclass correlation coefficient (ICC) and its 95% confidence interval (CI) were calculated using a two-way random effects model for absolute agreement using single measures [57]. ICC values are bound from 0 to 1, where values closer to 1 indicate stronger reliability. The following criteria were used to interpret ICC values and their 95% confident intervals: >0.9 excellent, 0.75–0.9 good, 0.5–0.75 moderate, and <0.5 poor [57]. The standard error of the measurement (SEM) was calculated according to the following equation: SEM = √within-participant variance from the mixed model [58].

For variables which failed the normality test, two separate analyses were carried out using (i) the original data and (ii) transformed data. The first analysis of non-normal data in its original form involved calculating an ICC using a generalized linear mixed model with Gamma distribution and identity link using the “lme4” package [59]. In addition to location (mean) and scale (variance) parameters, Gamma distribution also has a shape parameter that allows it to better fit skewed data. The ICC was then estimated using the methodology where observation-level variance is substituted for error variance [60]. SEMS were not calculated for data analysed with the gamma model. In the cases where a Gamma distribution could not be used to fit the data, a non-parametric measure of reliability was computed on the non-transformed data using Lin’s concordance correlation coefficient (CCC) [61,62] where values <0.9 were interpreted as indicative of poor repeatability [63]. The second analysis of non-normal data involved transforming the data using arcsin transformation to bring the variable closer to normality [64]. The ICC was then calculated on the transformed scale using the same procedure described for normally distributed data [57]. The fitness of the model to the data was evaluated using QQ-plots and residuals versus fitted values plots. See Supplementary Materials for a full description of the statistical analysis.

## 3. Results

### 3.1. Study Participants

Fifteen people with stroke participated in the research. Data for two participants were excluded due to failure to complete the protocol; one participant was unable to adequately follow instructions and the other experienced severe sleepiness. In addition, methodological errors resulted in the loss of one participant’s data. The remaining 12 participants (male *n* = 6, female *n* = 6) were an average age of 68.8 ± 11.0 years and 4.9 ± 3.8 years post stroke, and the majority had a left hemiparesis (*n* = 9). Participants presented with a range of lower limb impairment and used a variety of outdoor mobility aids (wheelchair *n* = 2, frame *n* = 2, quad stick *n* = 2, stick *n* = 2, and no aids *n* = 4). Two participants wore an ankle foot orthosis. The mean ankle dorsiflexion MVC across the two testing sessions was 145.4 ± 65.6 Newtons (N) (range 57.2–262.0 N).

### 3.2. Descriptive Results

The descriptive data for all variables can be seen in Table 1. There was a mean TA VA of 84.8 ± 19.7% for the ITT method, 86.9 ± 17.0% for the adjusted ITT method, and 96.2 ± 6.3% for CAR method.

### 3.3. Reliability Results

Refer to Table 1 for reliability results. The reliability of the twitch amplitudes used to calculate VA was as follows; the initial resting twitch had an ICC of 0.57 (95% CI 0.07 to 0.86) with the very low lower bound CI indicating poor reliability, whereas the initial superimposed twitch had an ICC of 0.83 (95% CI 0.55 to 0.95, based on transformed data) indicating reliability of a moderate level. For the twitch amplitudes used to calculate VA at the end of the task, the final superimposed twitch had an ICC of 0.65 (95% CI 0.19 to 0.88, based on transformed data) and the final resting twitch had an ICC of 0.73 (95% CI 0.31 to 0.92); for both twitches, the lower bound CIs fell in the poor range for reliability.

For the VA calculations based on the non-transformed data, the ICCs for VA^ITT^ and VA^Adj_ITT^ were both 0.62, but the lower bound CIs fell in the poor range (VA^ITT^ 95% CIs 0.25 to 0.74 and VA^Adj_ITT^ 95% CI 0.3 to 0.74), whereas the ICC for VA^CAR^ was 0.82, with a lower bound CI in the moderate range (VA^CAR^ 95% CI 0.63 to 0.91). For the ITT calculations using transformed data, the ICCs for VA^ITT^ and VA^Adj_ITT^ were both 0.82 with lower bound CIs in the moderate range (VA^ITT^ 95% CI 0.53 to 0.94 and VA^Adj_ITT^ 95% CI 0.51 to 0.94). The VA^CAR^ calculation using transformed data produced the highest reliability with an ICC of 0.92, and a lower bound CI in the good range (95% CI 0.77 to 0.98). In contrast to VA at the start of the task, VA calculated at the end of the task had poor reliability using both the ITT and CAR methods. The central fatigue data could not be fitted using a gamma distribution and the presence of negative values prevented transformation, but the CCCs demonstrated zero reliability of these parameters (CCCs < 0).

## 4. Discussion

This is the first study to assess the between-session reliability of VA of the TA muscle (using peripheral electrical stimulation) in any population, healthy or stroke. The findings demonstrate that VA using a doublet pulse over the hemiparetic TA muscle has between-session reliability ranging from good to poor depending on the calculation method and statistical analysis method.

### 4.1. Voluntary Activation

#### 4.1.1. Transformed versus Non-Transformed Data

For the analysis of transformed data, VA^CAR^ had an ICC in the excellent range with the lower bound 95% CI in the good range (VA^CAR^ ICC = 0.92, 95% CI 0.77 to 0.98). Whereas VA^ITT^ and VA^Adj_ITT^ had lower ICCs, with lower bound 95% CIs in the moderate range (see Table 1). For the analysis of non-transformed data, all ICCs were lower, but the CAR method was still superior to the ITT methods (VA^CAR^ ICC 0.82, 95% CI 0.63 to 0.91; VA^ITT^ ICC 0.62, 95% CI 0.25 to 0.74; VA^Adj_ITT^ ICC 0.62, 95% CI 0.30 to 0.74). Literature indicates that both the ICCs and their CIs should be considered when interpreting reliability data [57]. Therefore, the observation that lower-bound CIs for the ITT methods fell in the poor range is concerning and suggests less certainty about the reliability of these parameters when analyzing non-transformed data. The higher reliability for all VA calculations when using transformed data suggests researchers who are using VA as an outcome measure should consider transforming the data prior to conducting further analyses, for example, to determine the effect of an intervention. Data transformation offers a further advantage of allowing the application of traditional analysis of variance (ANOVA) models which assume normality of the data; however, the conversion to transformed-scale units makes interpretation difficult. For example, the mean effect of an intervention is of no use to researchers and clinicians when it is provided in log-scale units. For this reason, we have provided reliability analyses using both transformed and non-transformed methods, to allow researchers to choose the scale which better suits their needs.

#### 4.1.2. ITT versus CAR Calculations

Poorer reliability for the ITT method versus the CAR method is in agreement with the reliability findings for the healthy quadriceps muscle [20] and higher heterogeneity for the ITT method [16]. Increased variance in the ITT measure may relate to the use of the resting twitch in its calculation. Our findings showed a wide 95% CI for the ICC of the initial resting twitch amplitude (ICC 0.57, 95% CI 0.07 to 0.86), and as this parameter is included in the ITT calculation, it may have contributed to the lower ICC. We used a resting twitch that had been induced just prior to the MVC; however Place et al. [20] demonstrated that VA^ITT^ calculated using a potentiated resting twitch (induced 2 s following a brief MVC) was slightly more reliable than inducing the resting twitch before the MVC, although both methods were less reliable than the CAR method. The protocol in the present study did not permit the use of a potentiated resting twitch for VA^ITT^ (as the MVC was sustained for a further 30 s to measure central fatigue); however, this should be considered in future research. Other methods to reduce the variability of the resting twitch amplitude should also be considered, such as taking the average of three resting twitches [65].

In terms of the differences between the two ITT methods, we observed that the VA^ITT^ and VA^Adj_ITT^ produced comparable ICCs. The adjusted calculation was developed to account for occasions when the initial superimposed twitch is delivered at a submaximal MVC [43,55], and therefore could be particularly relevant for people with stroke who have altered motor unit recruitment [66] and decreased force steadiness [67]. For a number of our participants, the initial superimposed twitch was delivered at a force slightly lower than their maximum (based on the average of their three MVCs), and therefore the adjusted ITT calculation resulted in slightly higher VA values. The need for an adjusted equation could be resolved if the timing of electrical stimulation aligned more closely with the persons peak MVC. In this study, the stimulation was delivered with a manual trigger, but an automatic-triggering system might improve stimulation timing [2,68]. In addition, in the present study one dataset was lost due to inconsistencies in twitch delivery between sessions and automatic triggering may have prevented this. The reliability of such a method would need to be assessed in people with stroke; however, given the minimal effects of the adjusted ITT calculation on VA reliability in this study, automatic triggering may not produce any substantial change in the reliability of VA^ITT^.

#### 4.1.3. Voluntary Activation as an Outcome Measure

VA measures are prone to ceiling effects [20] and this was a factor for two of our participants who achieved VA^ITT^ and VAR^CAR^ of 100% on at least one test occasion, despite having mild to moderate dorsiflexor strength deficits. This ceiling effect is problematic when considering using VA as an outcome measure, as it limits the ability to show an intervention effect. This issue could be addressed by using a greater intensity of electrical stimulation (e.g., 140% rather than 120% intensity [38]), or by using a burst rather than a doublet pulse [17], both of which would produce a larger twitch force. Of course, this would come at the risk of increased pain [18]. The CAR method may be considered at greater risk of ceiling effects due its higher estimation of VA [18,20]. It has also been criticized for providing a less valid measure of VA as the MVC is produced by a number of synergists whereas the superimposed twitch is produced by just the stimulated muscle [69]. Thus, despite the CAR method demonstrating higher reliability than the ITT method, its lower validity may limit its use as an outcome measure. As this study was nested within a clinical trial which tested the within-session effect of a neuromodulatory intervention [32], we had the opportunity to compare the effects of the intervention on both VA^ITT^ and VA^CAR^. Interestingly, where a univariate analysis had shown an insignificant effect of the neuromodulatory intervention on VA^ITT^ (*p* = 0.06), the same analysis showed a significant effect on VA^CAR^ (*p* = 0.03). This non-significant *p*-value for the ITT data is likely due to its larger SEM and lower reliability. This demonstrates a further disadvantage of using the less reliable ITT method when testing the effects of an intervention.

### 4.2. Central Fatigue

While the statistical analysis of central fatigue was limited to non-parametric tests, the negative CCC suggests zero reliability for these measures. This may have been influenced by the reliability of the superimposed twitch at the end of the task, which had an ICC of 0.65 and a lower-bound 95% CI of 0.19 in the poor range (based on transformed data); this poor reliability is likely to have been influenced by greater force fluctuations at the end of the task due to fatigue or reduced effort. In contrast to our findings, Signal et al. [46] measured central fatigue following a 90-s maximal quadriceps contraction in people with stroke, and reported an ICC of 0.82, with a lower bound 95% in the moderate range (95% CI 0.51 to 0.94). However, they did note increased variability in VA^ITT^ from people with stroke compared to healthy participants, which might have been influenced by force fluctuations at the end of the task. The findings of the present study suggest that central fatigue of the hemiparetic TA muscle after a 30-s isometric contraction is not a reliable measurement and should not be used as an outcome measure.

### 4.3. Methodological Considerations

While direct comparisons with previous reliability studies should be undertaken with caution due to the range of techniques used to apply electrical stimulation and to calculate VA, our results appear comparable or slightly superior to those obtained for between-session reliability in the healthy plantar flexor muscles (ICCs 0.35 to 0.87, with 3/5 studies reporting ICCs > 0.82) [40,41,42,43,44]. The two healthy studies with poor to moderate ICCs suggested this was due to low between-participant variability, which lowers the ICC [43,44]; however, this was not a factor in the sample of participants with stroke in the present study. Our results are also comparable but slightly less reliable than those obtained by Signal et al. [46] for VA^ITT^ of the hemiparetic quadriceps muscle (ICC = 0.98, 95% CI 0.94–0.99). The higher ICC reported by Signal et al. [46] likely relates to differences in their method and statistical analysis. Importantly, they collected three VA measures with 3-min rests between each and calculated the ICC based on “average measures” this method produces a higher ICC compared to the “single measures” analysis used in this study. We therefore recommend that researchers consider measuring VA from an average of three measurements, but also take into account how this changes the time and energy burden on participants. Higher reliability in Signal et al.’s study could also relate to differences in their sample, or to differences between the TA and quadriceps muscles. Previous research in healthy participants has shown that the reliability of VA and the resting twitch of the triceps surae muscles is influenced by knee and ankle joint angles [43,70]. The ankle position in our study was based on the optimum position for producing dorsiflexion torque [71], however, given the changes in muscle properties at different joint angles, future researchers should consider whether altering the ankle position might produce more consistent data. To further look for sources of variability in our data, we inspected individual data and noted that the greatest within-participant variation for TA VA^ITT^ between the two testing sessions occurred in the two participants with the greatest ankle dorsiflexion strength; this was despite these participants producing relatively consistent MVC and VA^CAR^ measures. For one participant, this variation was attributed to high variability in the resting twitch amplitude, and therefore VA^CAR^ was not susceptible to this variation. For the other participant, the variation was attributed to the superimposed twitch being delivered at a lower percentage of MVC; the participant had reached a stable peak force but this was lower for Test 1 than Test 2, perhaps due to insufficient effort, natural variations in force output, or fear of receiving the electrical stimulation. This resulted in a larger superimposed twitch and significantly lowered VA^ITT^. The adjusted ITT method only accounted for this slightly; however, the VAR^CAR^ was only minimally affected, likely because the variation in the size of the superimposed twitch was only small in comparison to the participants large MVC. For both of these participants who demonstrated high VA^ITT^ variability between sessions, the variation might have been reduced by taking the average of three VA measurements, as done by Signal et al. [46]. In addition, a practice test might reduce variability associated with task novelty and insufficient effort. Further research is needed to test this method in a larger sample of people with stroke and to explore the reliability of both the ITT and CAR calculations in a range of muscles and joint positions in people with varying levels of impairment.

Researchers in this field should take note of other steps that were taken in this study to reduce the variability of the measurements [2] including the use of a rigid set up, sufficient gain in the amplifier to detect small twitches, and careful visual inspection of force data. The electrical stimulation was applied to the TA muscle rather than the deep common peroneal nerve due to the risk of stimulating the neighboring superficial peroneal nerve [72] and activating the antagonist peroneus longus and brevis. We were careful to ensure there was no activation of the synergist extensor digitorum longus and extensor hallucis longus muscles. Muscle stimulation was also preferred for comfort and has been shown to provide more reliable VA measurements than nerve stimulation [33].

### 4.4. Strengths and Limitations

As described previously, there is a dearth of reliability data for VA in the stroke population. In addition, reports of any VA data (means and SDs) for the TA muscle in people with stroke are also scarce. One study reported comparable TA VA^ITT^ levels to our study for seven participants with chronic stroke (mean TA VA 91 ± 17%, exclusive of one participant with 0% VA) [12]. Given the general lack of available TA VA data, this study offers a significant contribution to the field.

This study did not investigate within-session reliability, however other studies would suggest that within-session reliability is likely to be better than the between-session reliability observed in this study [33,36,73]. This study has not addressed the reliability of VA measured with TMS; while other studies have done so [16], TMS has poor feasibility in people with stroke [32], and therefore we recommend the muscle stimulation technique described here. This study was small which is typical of reliability studies in this field, but the heterogenous sample was representative of people with a wide range of lower limb disability following stroke. Within the sample of 15, data for two participants were excluded due to impairments that had not been picked up during screening and which impaired the participants’ ability to consistently complete the protocol. Thus, this study’s findings are only applicable to participants who can follow instructions and maintain attention throughout the protocol and cannot be generalized to people with more severe cognitive or communication impairments.

## 5. Conclusions

The findings demonstrated that the reliability of VA measures obtained using a doublet pulse over the TA muscle is influenced by both the calculation method, ITT or CAR, and the statistical analysis. The most reliable results were obtained using the CAR calculation on transformed data, which produced an ICC of 0.92, and a lower bound confidence interval in the good range (95% CI 0.77 to 0.98). The CAR calculation using non-transformed data, and the ITT and adjusted-ITT calculations using transformed data, both produced moderately-reliable results (ICCs 0.82, lower bound 95% CIs 0.51–0.63). The reliability of the ITT and adjusted-ITT calculations using non-transformed data demonstrated the poorest reliability with lower bound confidence intervals in the poor range (ICCs 0.62, lower bound 95% CIs 0.25–0.30). Central fatigue measured at the end of the 30-s maximal contraction was not reliable. These findings offer significant insight to researchers considering using these measures to gauge the neurophysiological effects of stroke rehabilitation interventions. Researchers should consider VA as a potential measure to assess the effects of rehabilitation interventions that increase the central drive to a muscle.

## Figures and Tables

**Table 1:** Results.

	Test 1 Mean (SD)	Test 2 Mean (SD)	Change in Means (SD)	Analysis: Original Units Model ICC or CCC (95% CI) SEM Where Applicable		Analysis: Arcsin Scale Units Model ICC (95% CI)	
**Twitch contraction time (CT)**							
Resting twitch CT (s)	0.156 (0.036)	0.147 (0.026)	0.010 (0.026)				
Superimposed twitch CT (s)	0.063 (0.039)	0.057 (0.041)	0.000 (0.020)				
Superimposed twitch CT end task	0.087 (0.025)	0.074 (0.039)	0.013 (0.025)				
Resting twitch CT end task	0.131 (0.023)	0.132 (0.021)	−0.001 (0.015)				
**Twitch amplitudes**							
Resting twitch amplitude (N)	26.87 (9.63)	27.64 (10.53)	−0.77 (9.34)	Gaussian ICC = 0.57 (0.07, 0.86) SEM = 6.6			
Superimposed twitch amplitude (N)	4.21 (5.18)	3.94 (4.68)	0.27 (3.36)	Non-parametric CCC = 0.77 (0.38, 0.93)		Gaussian ICC = 0.83 (0.55, 0.95)	
Superimposed twitch amplitude end task (N)	7.93 (6.65)	7.59 (7.18)	0.35 (7.03)	Gamma ICC = 0.35 (0.05, 0.45)		Gaussian ICC = 0.65 (0.19, 0.88)	
Resting twitch amplitude end task (N)	25.28 (9.64)	26.47 (10.22)	−1.19 (7.30)	Gaussian ICC = 0.73 (0.31, 0.92) SEM = 5.16			
**Voluntary activation (VA)**							
VA^ITT^ (%)	84.6 (19.4)	85.1 (20.1)	−0.50 (11.21)	Gamma ICC = 0.62 (0.25, 0.74)		Gaussian ICC = 0.82 (0.53, 0.94)	
VA^Adj_ITT^ (%)	86.2 (18.1)	87.5 (15.9)	−1.32 (9.60)	Gamma ICC = 0.62 (0.30, 0.74)		Gaussian ICC = 0.82 (0.51, 0.94)	
VA^CAR^ (%)	96.2 (6.1)	96.1 (6.5)	0.16 (1.88)	Gamma ICC = 0.82 (0.63, 0.91)		Gaussian ICC = 0.92 (0.77, 0.98)	
VA^ITT^ end task (%)	70.9 (18.8)	71.3 (32.1)	−0.34 (28.98)	Non-parametric CCC = 0.39 (−0.11, 0.74)		Arcsin transformation not possible	
VA^CAR^ end task (%)	90.5 (11.6)	88.3 (16.1)	2.21 (16.71)	Gamma ICC = 0.33 (−0.23, 0.51)		Gaussian ICC = 0.48 (0.00, 0.82)	
**Central fatigue (CF)**							
CF^ITT^ (%)	13.7 (22.4)	13.8 (20.0)	−0.16 (33.05)	Non-parametric CCC = −0.22 (−0.68, 0.38)		Arcsin transformation not possible	
CF^CAR^ (%)	5.7 (12.3)	7.8 (10.7)	−2.05 (16.60)	Non-parametric CCC = −4.1 (−0.57, 0.51)		Arcsin transformation not possible	

SEM = standard error of measurement; CT = contraction time; VA^ITT^ = voluntary activation calculated using ITT method; VA^Adj_ITT^ = voluntary activation calculated using adjusted ITT method; VA^CAR^ = voluntary activation calculated using CAR method, CF^ITT^= central fatigue calculated using ITT method; CF^CAR^ = central fatigue calculated using CAR method.

## Supplementary Materials

The statistical analysis is available online at https://www.mdpi.com/2076-3425/11/2/176/s1.
