# Supplementary material for: Reliability of Tibialis Anterior Muscle Voluntary Activation Using the Interpolated Twitch Technique and the Central Activation Ratio in People with Stroke
Source: Brain Sci. 2021 Feb 1;11(2):176. doi: 10.3390/brainsci11020176 (PMC7912757; doi:10.3390/brainsci11020176)
Supplement: Supplementary file 1 [file brainsci-11-00176-s001.pdf]

Reliability Analysis

Usman Rashid

13/04/2020

Contents

1 Statistical Analysis Plan **2**

2 Summary Statistics **3**

2.1 Data Variables 3
2.2 Normality Test 3
2.3 ICC Calculations 3
2.3.1 Reliability on the Original Scale 3
2.3.2 Non-parametric Concordance Correlation Coefficient 4
2.3.3 Reliability on the Arcsin Transformed Scale 4
2.3.3.1 Goodness-of-the-fit plots for the Model 5

References **11**

1 Statistical Analysis Plan

Data is imported into R (R Core Team, 2018) for reliability analysis. The normality of the variables is checked with Shapiro-Wilk test. For variables which are normally distributed, a linear mixed model with Gaussian distribution and identity link is setup to estimate between-participant (σ_p^2), between-test (σ_t^2) and error (within-participant, σ_ϵ^2) variance using the *rptR* package (Stoffel, Nakagawa, & Schielzeth, 2017). An ICC for test-retest reliability with absolute agreement using single measures is calculated as described in the following equation (Koo & Li, 2016). The confidence interval for the ICC is constructed using 1000 parametric bootstraps.

$$ICC(A,1) = \frac{\sigma_p^2}{\sigma_p^2 + \sigma_t^2 + \sigma_\epsilon^2} \tag{1}$$

Variables which fail the normality test, two analyses are conducted. First, for an estimate of the ICC on the original scale, a generalised linear mixed model is setup with Gamma distribution and identity link using the *lme4* package (Bates, Mächler, Bolker, & Walker, 2015). In addition to location (mean) and scale (variance) parameters, Gamma distribution also has a shape parameter allowing it to better fit skewed data. ICC(A, 1) is estimated using the methodology described by Nakagawa, Johnson, & Schielzeth (2017) where observation-level variance is substituted for error variance (σ_ϵ^2) in equation 1. The observation-level variance is estimated from the fitted model as follows:

$$\sigma_\epsilon^2 = \frac{\beta_0^2}{\nu} \tag{2}$$

where β_0 is the overall intercept in the fitted model and ν is the shape parameter of the fitted Gamma distribution. In the cases where Gamma distribution cannot be used to fit the data, a non-parametric measure of reliability is computed with Lin's concordance correlation coefficient (Steichen & Cox, 2002), (Lawrence & Lin, 1989). The concordance coefficient quantifies the deviation of the observed data from the line of perfect concordance (a line at 45°).

Second, arcsin transformation is used when possible to bring the variable closer to normality. ICC(A, 1) is calculated on the transformed scale using the same procedure described earlier for normal data. The fitness of model to the data is evaluated using QQ-plot and residuals versus fitted values plot.

Discussion: The limitation of the transformed scale is that it is generally harder to interpret although it allows application of traditional ANOVAs which assume normality of the data for statistical inference. The advantage of reporting reliability for both the transformed scale and the original scale using two different approaches is that future researchers can choose the scale which better suits their needs.

2 Summary Statistics

2.1 Data Variables

```
## 'data.frame':    24 obs. of  13 variables:
##  $ PID         : Factor w/ 12 levels "1","2","3","4",..: 1 2 3 4 5 6 7 8 9 10 ...
##  $ Test        : Factor w/ 2 levels "1","2": 1 1 1 1 1 1 1 1 1 1 ...
##  $ ITT_1       : num  84 59.1 100 85.5 93.6 ...
##  $ ITTAdj_1    : num  84.4 67.2 100 88.3 94.4 ...
##  $ CAR_1       : num  97.5 86.3 100 98.7 98.7 ...
##  $ ITT_2       : num  57.1 50.2 91.1 81.4 34 ...
##  $ CAR_2       : num  89.2 86.6 97.5 98.6 57 ...
##  $ CF_ITT      : num  26.95 8.91 8.91 4.1 59.54 ...
##  $ CF_CAR      : num  8.2241 -0.3125 2.4516 0.0752 41.7339 ...
##  $ Rest_Tw_AMP_1: num  42.6 24.2 25.6 21 30.9 ...
##  $ Sup_Tw_AMP_1 : num  6.81 9.91 0 3.05 1.99 ...
##  $ Sup_Tw_AMP_2 : num  17.39 10.28 2 3.17 21.74 ...
##  $ Rest_Tw_AMP_2: num  40.5 20.6 22.5 17 32.9 ...
```

2.2 Normality Test

Variable	W.statistic	P.Value	Sig.
ITT_1	0.7566365	0.0000624	TRUE
ITTAdj_1	0.7668580	0.0000887	TRUE
CAR_1	0.6324977	0.0000015	TRUE
ITT_2	0.8798619	0.0082571	TRUE
CAR_2	0.7325573	0.0000280	TRUE
CF_ITT	0.9634983	0.5126192	FALSE
CF_CAR	0.7745614	0.0001163	TRUE
Rest_Tw_AMP_1	0.9603490	0.4454757	FALSE
Sup_Tw_AMP_1	0.7904843	0.0002067	TRUE
Rest_Tw_AMP_2	0.9657427	0.5639649	FALSE
Sup_Tw_AMP_2	0.8989557	0.0204552	TRUE

2.3 ICC Calculations

2.3.1 Reliability on the Original Scale

Fitting a model with Gamma distribution is not possible for ITT_2 and Sup_Tw_AMP_1 as these contain 0 or negative values. For CF_ITT and CF_CAR, the models failed to converge with a non-negative between-participant variance.

Note: The SEMs presented in the below table are on the original scale.

Variable	Model	ICC	CI.lower	CI.upper	SEM
ITT_1	Gamma	61.5	25.4	73.8	
ITTAdj_1	Gamma	62.2	30.3	74.1	
CAR_1	Gamma	82.1	63.1	90.7	
CAR_2	Gamma	32.6	-23.3	51.0	
Rest_Tw_AMP_1	Gaussian	57.0	6.6	85.6	6.607
Rest_Tw_AMP_2	Gaussian	72.7	31.0	91.9	5.160
Sup_Tw_AMP_2	Gamma	35.4	5.0	44.7	

2.3.2 Non-parametric Concordance Correlation Coefficient

Negative values for reliability suggest that the participants are more different than the average similarity level obtained from random measurements (Stoffel et al., 2017). In other words, negative values are an evidence of zero (poor) reliability.

Variable	CCC	CI.lower	CI.upper
ITT_2	39.3	-11.0	73.5
CF_ITT	-21.5	-68.3	37.8
CF_CAR	-4.1	-56.8	50.9
Sup_Tw_AMP_1	76.6	38.0	92.5

2.3.3 Reliability on the Arcsin Transformed Scale

The variables are transformed as follows (Warton & Hui, 2011):

$$X_t = asin\left(\sqrt{\frac{X_o}{100}}\right) \tag{3}$$

Arcsin transformation is not possible for ITT_2, CF_ITT and CF_CAR as these variables contain negative values for which arcsin is not defined.

Note: The SEMs presented in the below table are for the transformed scale.

Variable	ICC	CI.lower	CI.upper	SEM
ITT_1	82.2	53.3	93.7	0.117
ITTAdj_1	82.2	51.1	94.3	0.105
CAR_1	92.4	77.1	97.6	0.039
CAR_2	48.3	0.0	81.6	0.153
Sup_Tw_AMP_1	83.1	54.7	94.9	0.053
Sup_Tw_AMP_2	64.7	19.2	87.9	0.082

2.3.3.1 Goodness-of-the-fit plots for the Model

Arcsin transformed CAR_2 has a larger number of outliers compared to the other measures.

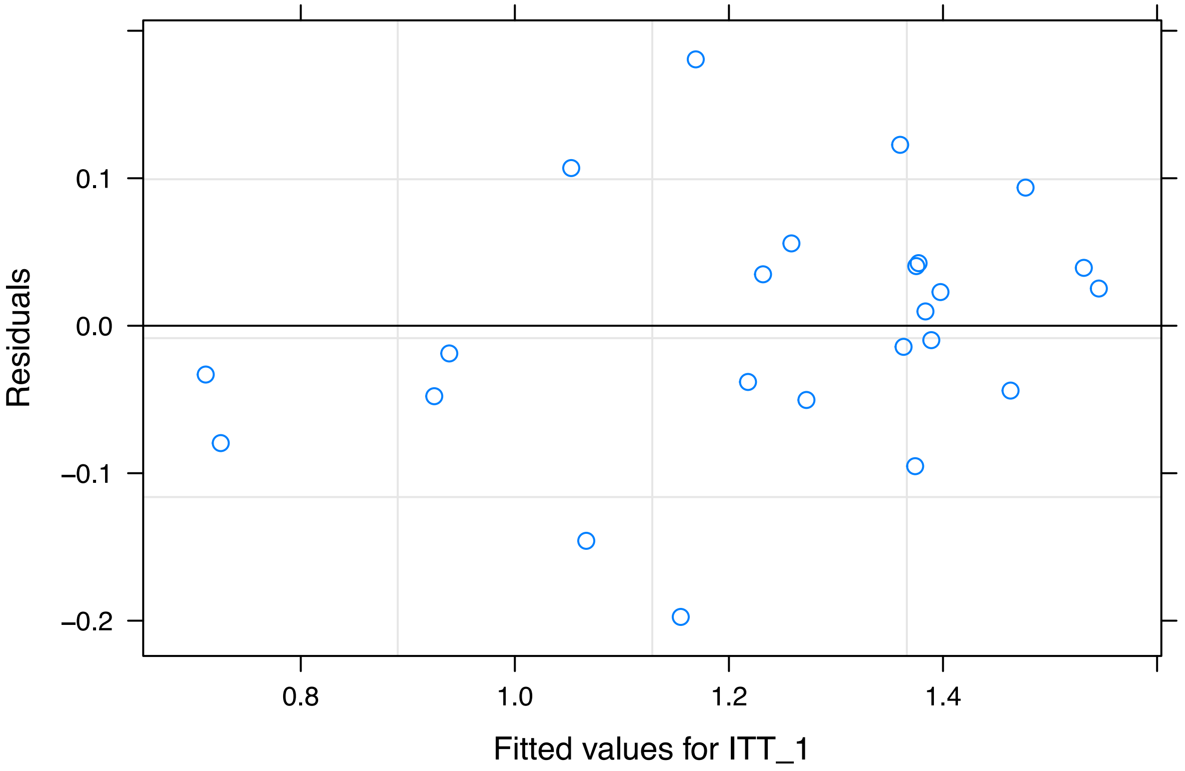

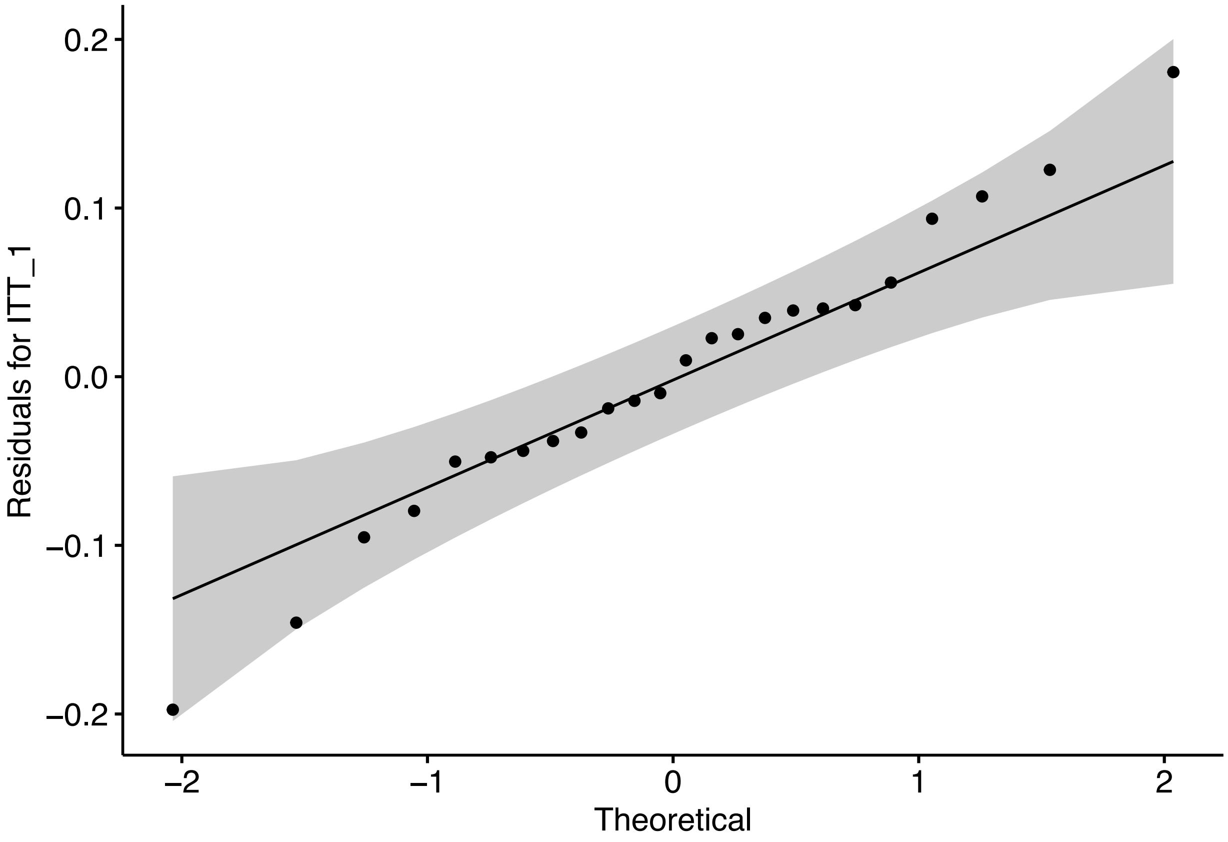

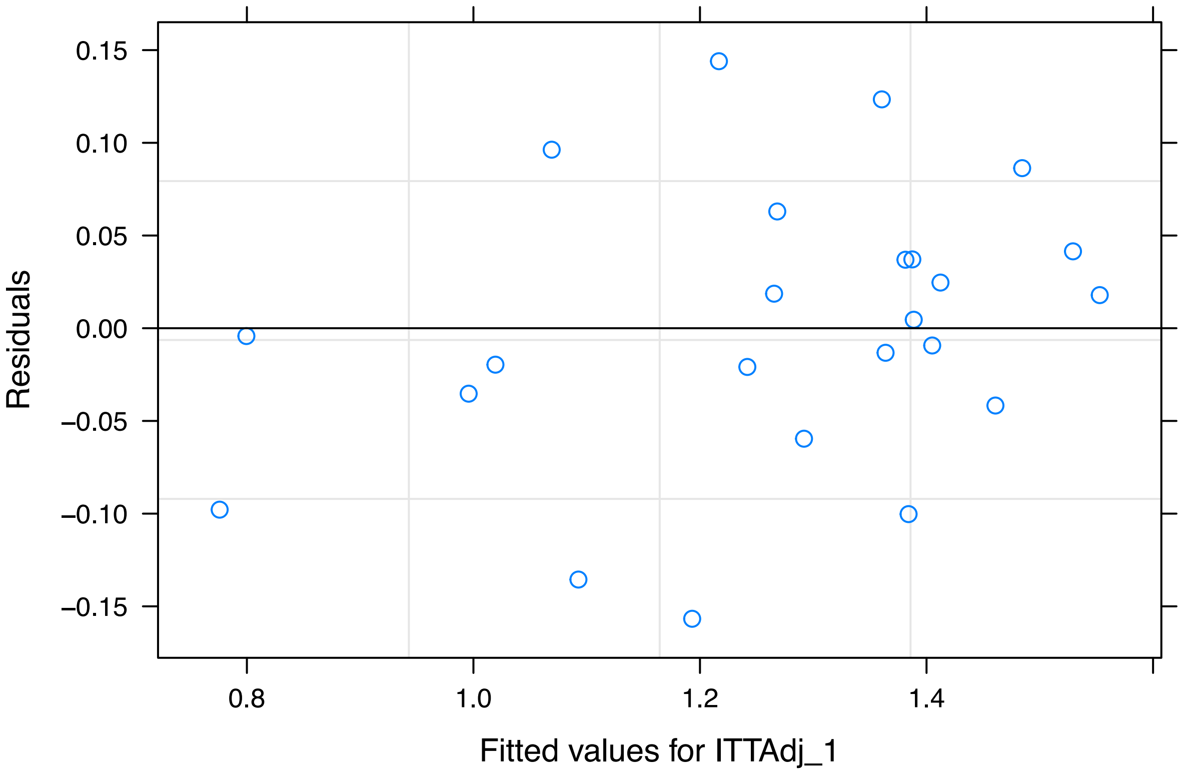
Residuals
Fitted values for ITTAdj_1
0.15
0.10
0.05
0.00
−0.05
−0.10
−0.15
0.8
1.0
1.2
1.4

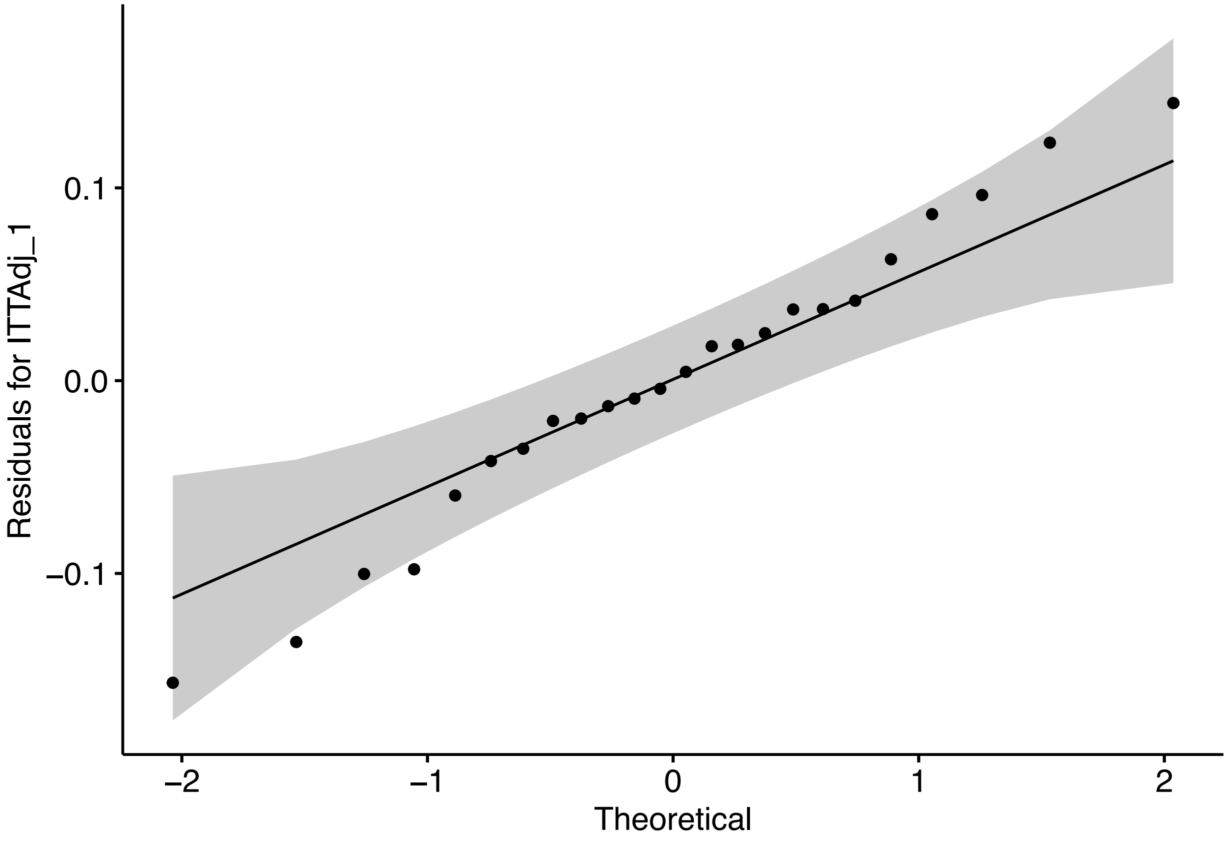
Residuals for ITTAdj_1
Theoretical
0.1
0.0
−0.1
−2
−1
0
1
2

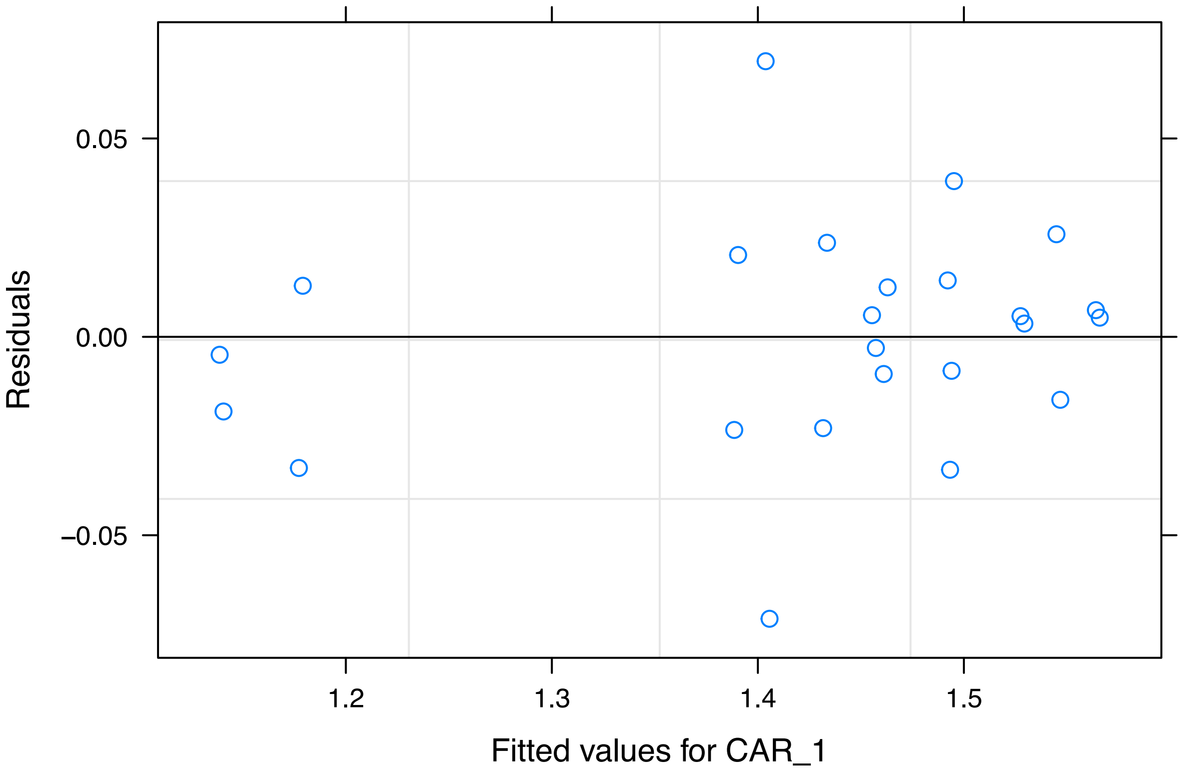
0.05
0.00
−0.05
Residuals
1.2
1.3
1.4
1.5
Fitted values for CAR_1

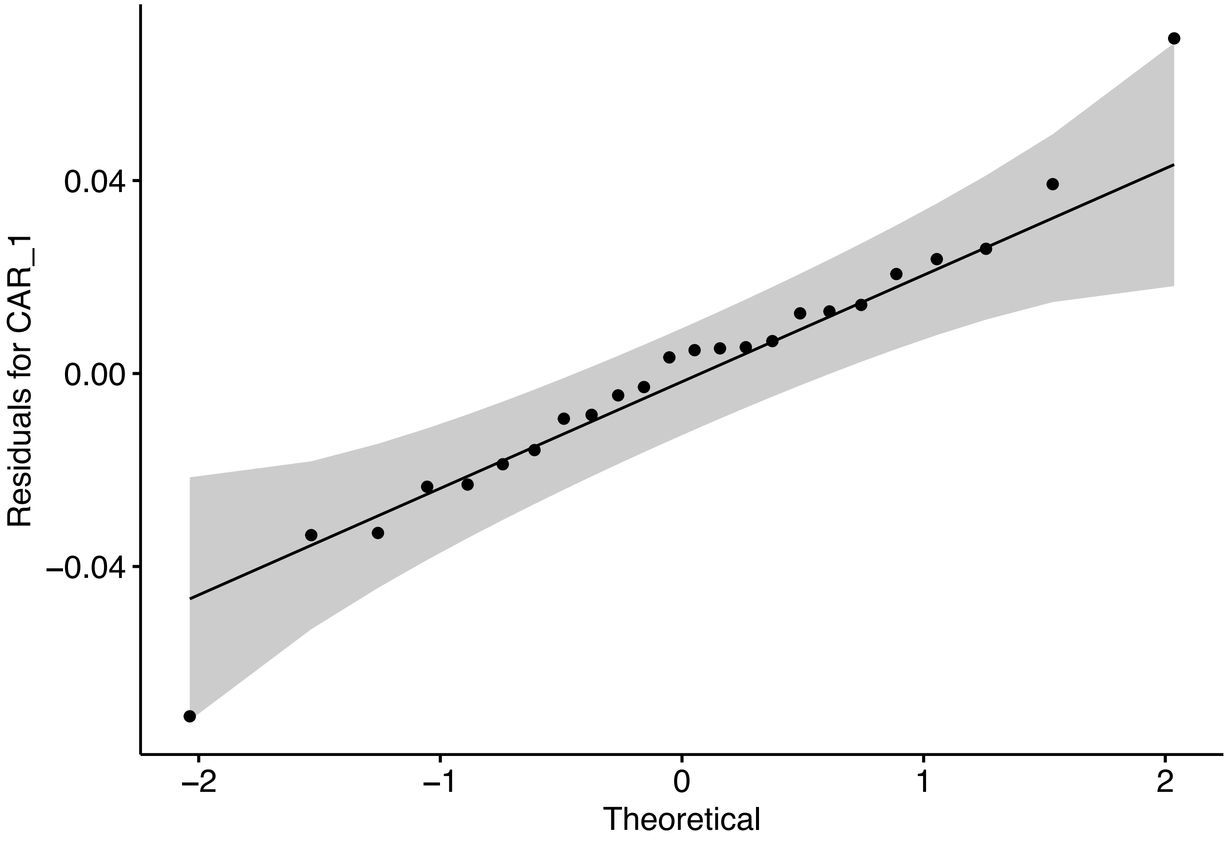
0.04
0.00
−0.04
Residuals for CAR_1
−2
−1
0
1
2
Theoretical

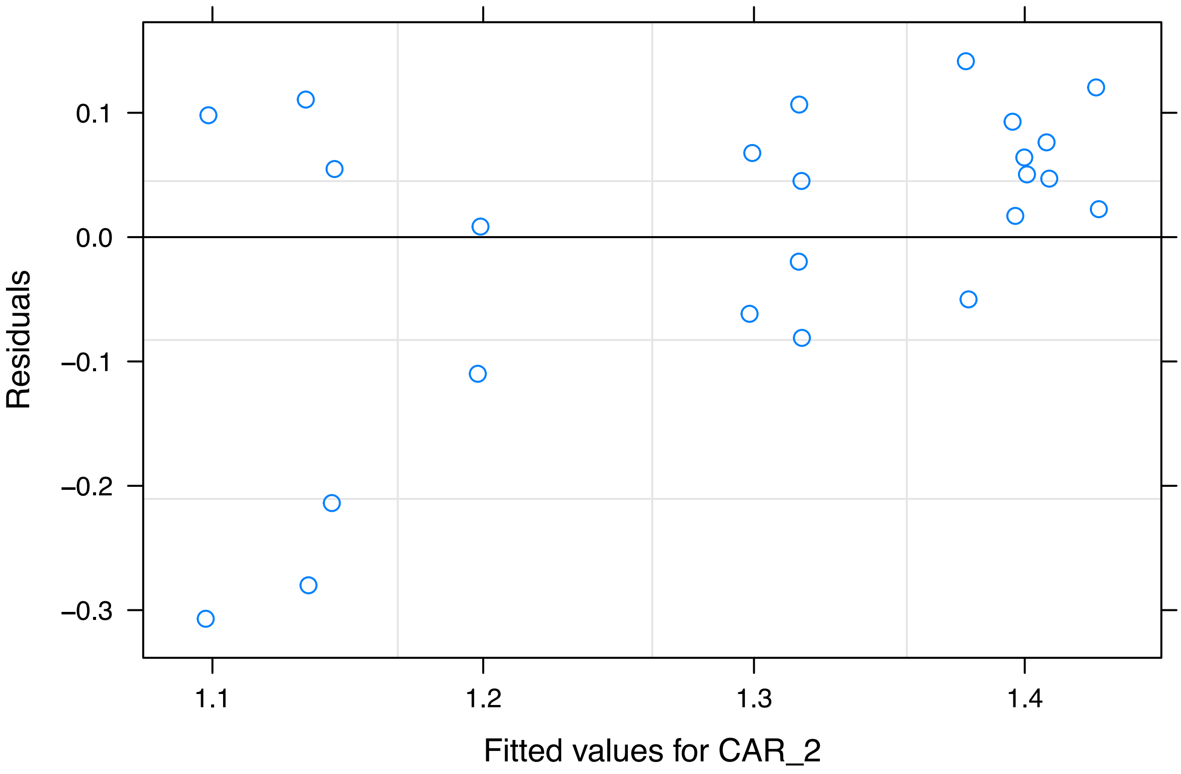

0.1
0.0
−0.1
−0.2
−0.3
Residuals
1.1
1.2
1.3
1.4
Fitted values for CAR_2

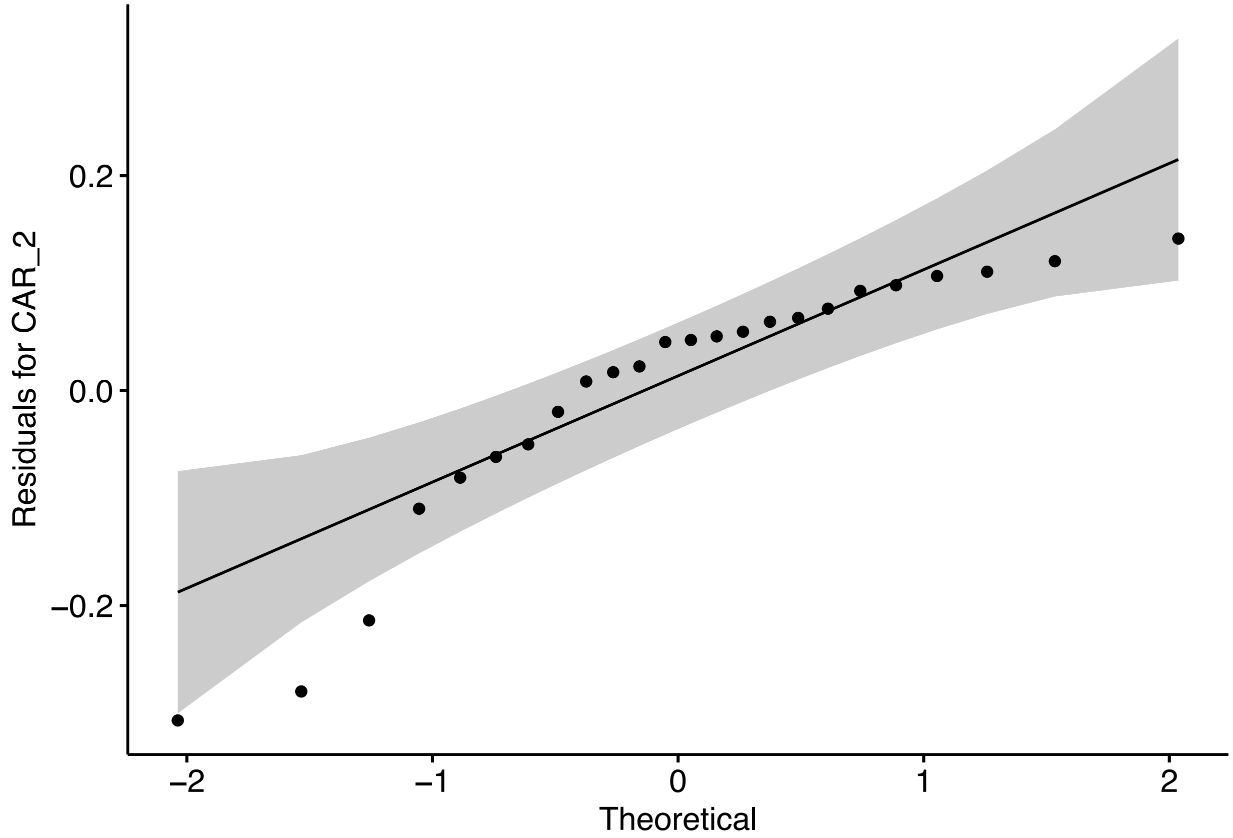

0.2
0.0
−0.2
Residuals for CAR_2
−2
−1
0
1
2
Theoretical

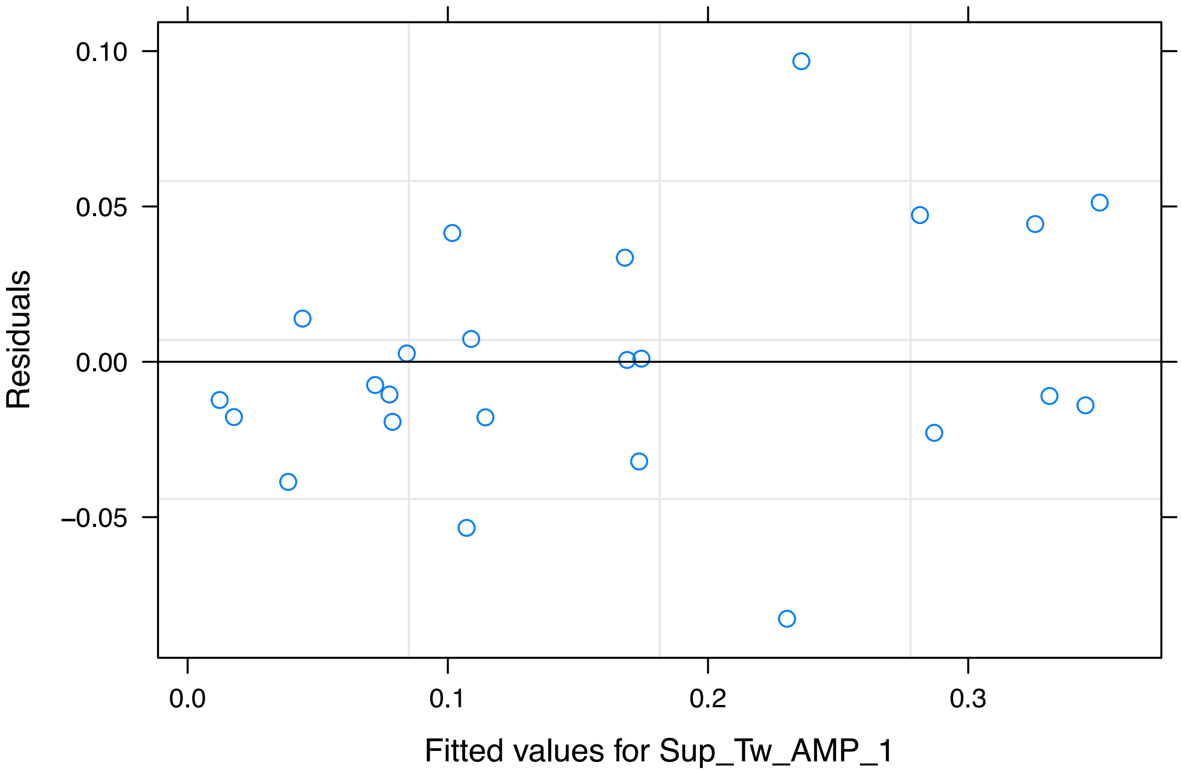

0.10
0.05
0.00
−0.05
Residuals
0.0
0.1
0.2
0.3
Fitted values for Sup_Tw_AMP_1

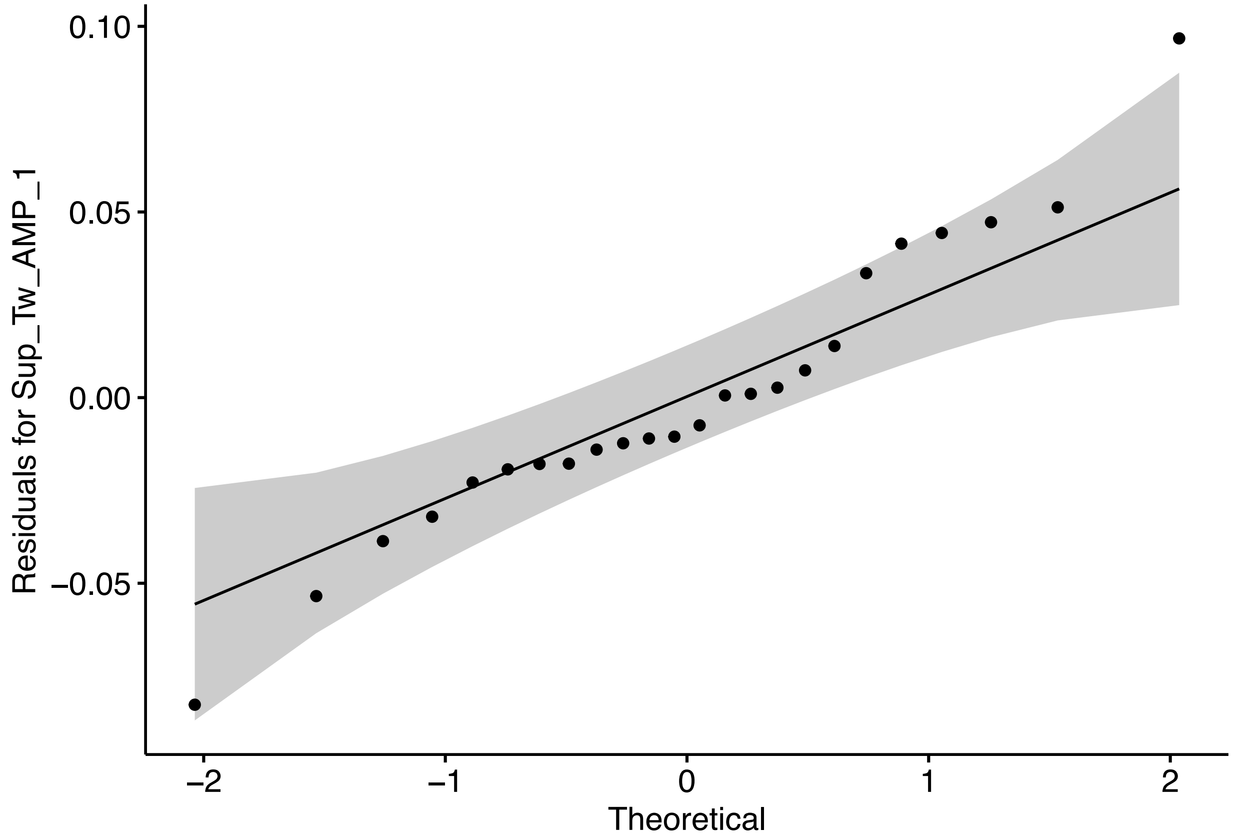

0.10
0.05
0.00
−0.05
Residuals for Sup_Tw_AMP_1
−2
−1
0
1
2
Theoretical

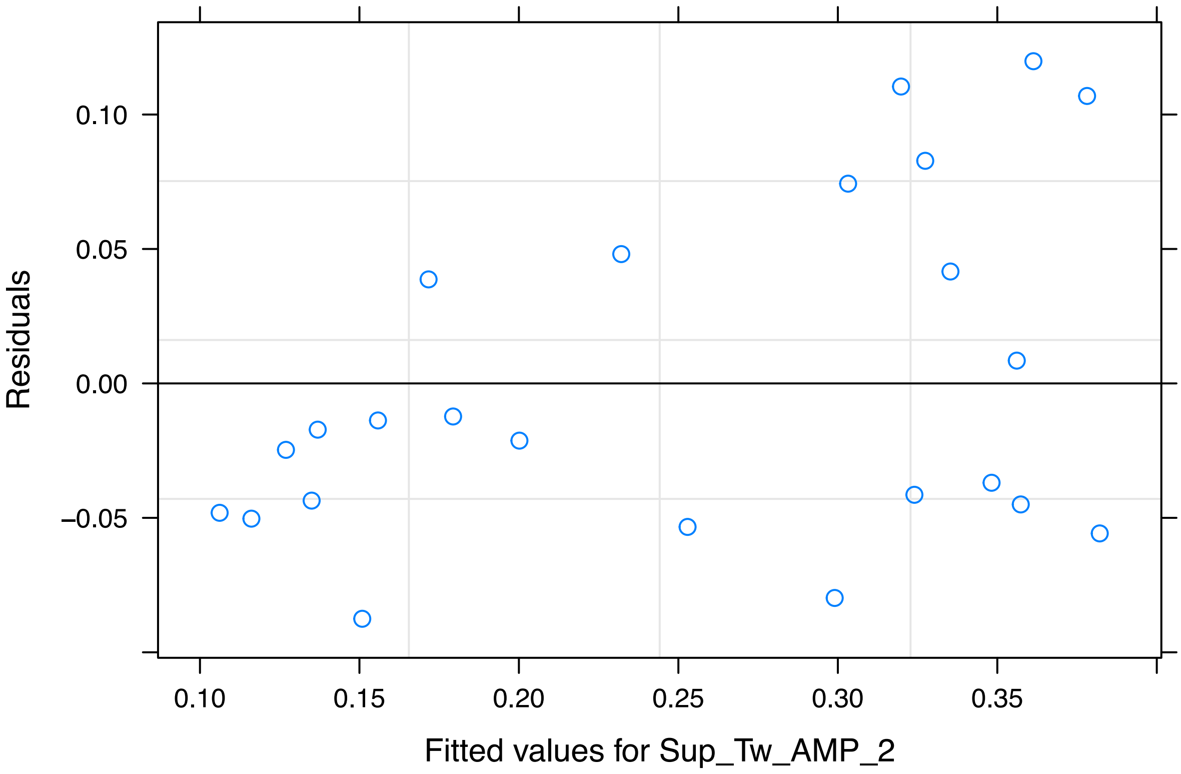
0.10
0.05
0.00
−0.05
Residuals
0.10
0.15
0.20
0.25
0.30
0.35
Fitted values for Sup_Tw_AMP_2

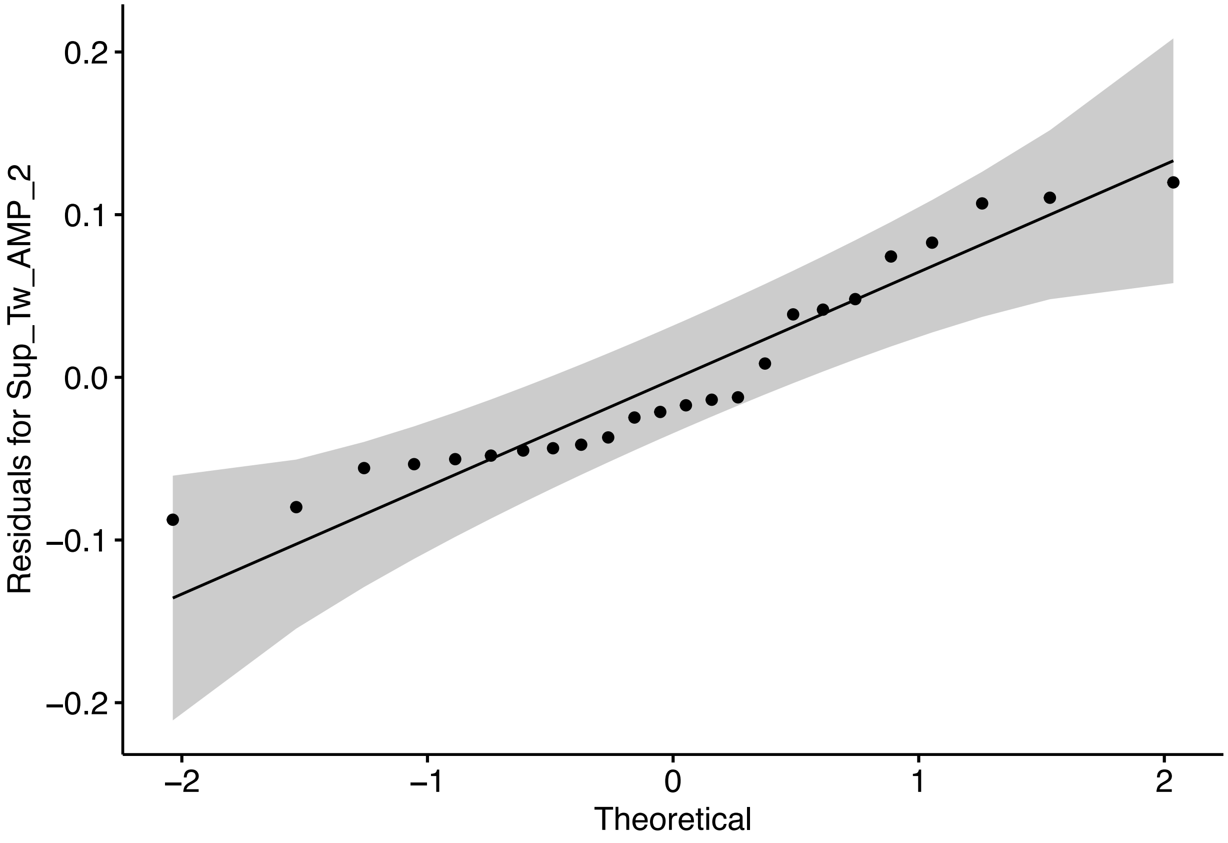
0.2
0.1
0.0
−0.1
−0.2
Residuals for Sup_Tw_AMP_2
−2
−1
0
1
2
Theoretical

References

Bates, D., Mächler, M., Bolker, B., & Walker, S. (2015). Fitting linear mixed-effects models using lme4. *Journal of Statistical Software*, *67*(1), 1–48. https://doi.org/10.18637/jss.v067.i01

Koo, T. K., & Li, M. Y. (2016). A guideline of selecting and reporting intraclass correlation coefficients for reliability research. *Journal of Chiropractic Medicine*, *15*(2), 155–163.

Lawrence, I., & Lin, K. (1989). A concordance correlation coefficient to evaluate reproducibility. *Biometrics*, 255–268.

Nakagawa, S., Johnson, P. C., & Schielzeth, H. (2017). The coefficient of determination r 2 and intra-class correlation coefficient from generalized linear mixed-effects models revisited and expanded. *Journal of the Royal Society Interface*, *14*(134), 20170213.

R Core Team. (2018). *R: A language and environment for statistical computing.* Retrieved from https://www.R-project.org/

Steichen, T. J., & Cox, N. J. (2002). A note on the concordance correlation coefficient. *The Stata Journal*, *2*(2), 183–189.

Stoffel, M. A., Nakagawa, S., & Schielzeth, H. (2017). RptR: Repeatability estimation and variance decomposition by generalized linear mixed-effects models. *Methods in Ecology and Evolution*, *8*(11), 1639???1644. https://doi.org/10.1111/2041-210X.12797

Warton, D. I., & Hui, F. K. (2011). The arcsine is asinine: The analysis of proportions in ecology. *Ecology*, *92*(1), 3–10.
